# Documentation of Adverse Social Drivers of Health in US Emergency Departments

**DOI:** 10.1016/j.acepjo.2025.100272

**Published:** 2025-10-29

**Authors:** Melanie F. Molina, Rebecca E. Cash, Janice A. Espinola, Krislyn M. Boggs, Carlos A. Camargo, Margaret E. Samuels-Kalow

**Affiliations:** 1 Department of Emergency Medicine, University of California, San Francisco, California, USA; 2 Philip R. Lee Institute for Health Policy Studies, University of California, San Francisco, California, USA; 3 Department of Emergency Medicine, Massachusetts General Hospital, Harvard Medical School, Boston, Massachusetts, USA

## Abstract

**Objectives:**

This study aimed to characterize emergency department (ED)-based policies related to documentation of adverse social drivers of health (aSDOH) among a national sample of US EDs.

**Methods:**

We conducted a cross-sectional survey of ED leaders from a 5% stratified random sample of US EDs (*n* = 280) from January to October 2023. For EDs reporting written screening policies for core aSDOH (housing, food, transportation, and utilities) or regulatory requirement-driven screening (intimate partner violence, other violence, substance use, and mental health), we assessed documentation policies, including personnel responsible and documentation methods. Using survey-weighted analyses and multivariable logistic regression, we examined associations between documentation policies and ED characteristics (practice setting, urbanicity, visit volume, and 24/7 social work availability).

**Results:**

Of 232 responding EDs (83% response rate), 213 (93.2%, 95% CI, 89.3-95.7) reported screening for at least 1 risk factor. Among these, 196 (93.8%, 95% CI, 87.9-96.9) had policies requiring documentation of positive screens. Documentation requirements were higher for regulatory requirement-driven screening (84-93.5) compared with core aSDOH screening (56.8-68.9). Clinical providers most commonly documented findings (97.8%, 95% CI, 94.8-99.1), followed by social workers/care coordinators (52.6%, 95% CI, 42.4-62.6). All documentation occurred in the electronic health record. In adjusted analyses, 24/7 social work availability was associated with greater odds of having at least 1 aSDOH documentation policy (odds ratio, 2.86; 95% CI, 1.09-7.52).

**Conclusion:**

Although most EDs with screening policies required documentation of positive screens, requirements varied by domain. Future research should focus on standardizing documentation practices and developing systems to effectively translate documented social needs into meaningful interventions.

**The Bottom Line**

How do emergency departments document social needs such as housing instability and food insecurity when identified during screening? In this national survey of US emergency departments, we found that 94% of departments that screen for social needs required documentation, primarily by clinical providers, with all documentation occurring in the electronic health record. Documentation was more common for regulatory-required screenings than for other social needs, and departments with 24/7 social workers were nearly 3 times more likely to have documentation policies. We need standardized approaches for capturing social needs in electronic health records to facilitate meaningful interventions.

## 1 Introduction

### 1.1 Background and Importance

Electronic health record (EHR)-based provider documentation of adverse social drivers of health (aSDOH) can be used to inform treatment, interventions, and even risk-adjusted insurance payments. In 2019, the National Academies of Sciences, Engineering, and Medicine emphasized the importance of EHR-based aSDOH data in integrating social and medical care. However, they also recognized a lack of best-practice documentation standards, which are integral to translating aSDOH information into meaningful response.^1^ This lack of standards has likely contributed to underdocumentation of both structured and unstructured aSDOH data across multiple domains, including housing, food, employment, and education.^2^ The problem is particularly evident in emergency departments (EDs),^3^ in which patients have a high prevalence of aSDOH.^4^

### 1.2 Goals of This Investigation

Currently, little is known about national ED-based aSDOH documentation practices. We therefore sought to characterize ED-based policies related to aSDOH documentation among a national sample of US EDs.

## 2 Methods

From January to October 2023, we conducted a cross-sectional study of a 5% stratified random sample of 280 US ED leaders from the National Emergency Department Inventory (NEDI)-USA. If EDs reported having written screening policies for core aSDOH (housing instability, food insecurity, transportation difficulties, and trouble paying for utilities) or other regulatory requirement-driven screening (intimate partner violence [IPV], other exposure to violence, substance use, and/or mental health), we subsequently asked about written aSDOH documentation policies, specifically (1) who documented (eg, registration staff and social worker) and (2) the method of documentation (eg, recorded in the EHR and local hospital database) (eMethods). The Mass General Brigham Institutional Review Board classified this work as exempt. We followed the STROBE reporting guidelines.

We compared documentation policies by screening status, domain, and documentation method using survey-weighted proportions with 95% CIs. We explored the association of ED characteristics (practice setting, urbanicity, visit volume, and 24/7 availability of social work services) that we hypothesized to be associated with the presence of at least 1 aSDOH documentation policy as a binary outcome using survey-weighted multivariable logistic regression models. Analyses were performed using Stata 18.0 (Stata Corp).

## 3 Results

Of the 280 EDs surveyed, 232 (83%) responded to the screening policy questions, representing approximately 4% of the 5622 EDs in the NEDI-USA sample in 2022. The demographics of responding EDs are described elsewhere.^5^ Of 213 EDs (93.2%, 95% CI, 89.3% to 95.7%) reporting screening policies for at least 1 risk factor, 196 (93.8%, 95% CI, 87.9% to 96.9%) had policies requiring documentation of positive screens. Eighty-four to 93.5% of EDs with regulatory requirement-driven screening policies required documentation; 56.8% to 68.9% of EDs with screening policies for aSDOH required documentation (Table 1). Positive screens were most often documented by clinical providers (97.8%, 95% CI, 94.8% to 99.1%), followed by social workers or care coordinators (52.6%, 95% CI, 42.4% to 62.6%). All were recorded in the EHR (Table 2). In multivariable analysis adjusting for practice setting (academic vs community), urbanicity, and visit volume, 24/7 availability of social work services was associated with greater odds of having at least 1 aSDOH documentation policy (odds ratio [OR] 2.86, 95% CI, 1.09-7.52) (Table 3).

**Table 1:** Presence of policies requiring documentation of unmet social needs among emergency departments with screening policies (*n* = 213), by need.

Social need screening category	Total	Required documentation		Did not require documentation	
		*n*^a^	Weighted row % (95% CI)	n	Weighted row % (95% CI)
Housing instability or homelessness	64	44	68.4 (49.6-82.7)	20	31.6 (17.3-50.4)
Food insecurity or hunger	45	31	68.9 (44.5-86.0)	14	31.1 (14.0-55.5)
Difficulty obtaining transportation	41	24	56.8 (34.8-76.5)	17	43.2 (23.5-65.2)
Trouble paying for utilities	14	8	67.9 (32.6-90.3)	6	32.1 (9.8-67.4)
Intimate partner violence	179	148	84.8 (75.4-91.0)	31	15.2 (9.0-24.7)
Other exposure to violence	146	121	85.5 (74.9-92.1)	25	14.5 (8.0-25.1)
Substance use	170	135	80.0 (70.0-87.3)	35	20.0 (12.7-30.0)
Mental health	204	188	93.5 (87.3-96.8)	16	6.5 (3.2-12.7)

CI, confidence interval.

a Unweighted frequencies are shown.

**Table 2:** Policies requiring documentation of unmet needs among emergency departments with screening policies, by need.

	Any risk factor^a^ (*n* = 196)^b^	Housing instability (*n* = 59)	Food insecurity (*n* = 41)	Transportation difficulties (*n* = 37)	Trouble paying for utilities (*n* = 13)	Intimate partner violence (*n* = 169)	Other exposure to violence (*n* = 139)	Substance use (*n* = 160)	Mental health (*n* = 193)
	Weighted column % (95% CI)	Weighted column % (95% CI)	Weighted column % (95% CI)	Weighted column % (95% CI)	Weighted column % (95% CI)	Weighted column % (95% CI)	Weighted column % (95% CI)	Weighted column % (95% CI)	Weighted column % (95% CI)
**Who documents needs?**									
Registration staff									
Yes	7.3 (3.4-15.2)	8.0 (2.7-21.3)	2.7 (0.7-9.7)	3.1 (0.8-11.1)	0.0	8.3 (3.8-17.2)	9.0 (4.1-18.8)	7.9 (3.5-16.9)	7.5 (3.4-15.5)
No	92.7 (84.8-96.7)	92.0 (78.7-97.3)	97.3 (90.3-99.3)	96.9 (88.9-99.2)	100.0	91.7 (82.8-96.2)	91.0 (81.2-95.9)	92.1 (83.2-96.5)	92.6 (84.5-96.6)
Social worker/care coordinator									
Yes	52.6 (42.4-62.6)	70.9 (52.0-84.5)	60.4 (34.9-81.3)	90.2 (71.2-97.2)	71.2 (33.2-92.5)	52.5 (41.7-63.1)	56.0 (44.2-67.1)	53.7 (42.8-64.3)	53.7 (43.4-63.7)
No	47.4 (37.4-57.6)	29.1 (15.5-48.0)	39.6 (18.7-65.1)	9.8 (2.9-28.8)	28.8 (7.5-66.9)	47.5 (36.9-58.3)	44.0 (32.9-55.8)	46.3 (35.7-57.2)	46.3 (36.3-56.6)
Clinical provider (RN/APP, resident, attending)									
Yes	97.8 (94.8-99.1)	97.0 (81.2-99.6)	95.2 (72.0-99.4)	91.1 (69.7-97.9)	82.3 (34.7-97.6)	98.2 (94.7-99.4)	97.6 (93.7-99.1)	98.0 (94.4-99.3)	97.7 (94.7-99.1)
No	2.2 (0.9-5.2)	3.0 (0.4-18.8)	4.8 (0.6-28.0)	8.9 (2.2-30.3)	17.7 (2.4-65.4)	1.8 (0.6-5.3)	2.4 (0.9-6.4)	2.0 (0.7-5.6)	2.3 (0.9-5.4)
Entered directly by patient/guardian									
Yes	3.5 (1.0-11.1)	3.0 (0.4-18.8)	4.8 (0.6-28.0)	5.5 (0.7-31.2)	17.7 (2.4-65.4)	3.9 (1.2-12.6)	4.4 (1.3-13.9)	3.9 (1.1-12.5)	3.5 (1.0-11.4)
No	96.6 (88.9-99.0)	97.0 (81.2-99.6)	95.2 (72.0-99.4)	94.5 (68.8-99.3)	82.3 (34.7-97.6)	96.1 (87.4-98.9)	95.6 (86.1-98.7)	96.1 (87.5-98.9)	96.5 (88.7-99.0)
Case manager									
Yes	2.1 (0.4-10.5)	0.0	0.0	0.0	0.0	0.0	2.2 (0.3-14.4)	2.0 (0.3-13.0)	2.2 (0.4-10.7)
No	97.9 (89.5-99.6)	100.0	100.0	100.0	100.0	100.0	97.8 (85.7-99.7)	98.0 (87.1-99.7)	97.8 (89.4-99.6)
**How are needs documented?**									
Recorded in the electronic health record									
Yes	100.0	100.0	100.0	100.0	100.0	100.0	100.0	100.0	100.0
No	0.0	0.0	0.0	0.0	0.0	0.0	0.0	0.0	0.0
Recorded in the local hospital database (separate from clinical health record)									
Yes	4.3 (1.6-11.1)	1.6 (0.2-10.5)	2.5 (0.3-16.3)	2.9 (0.4-18.5)	0.0	4.4 (1.5-12.4)	5.4 (2.0-13.9)	4.8 (1.8-12.4)	4.4 (1.6-11.3)
No	95.7 (88.9-98.4)	98.5 (89.5-99.8)	97.5 (83.7-99.7)	97.1 (81.5-99.6)	100.0	95.6 (87.7-98.5)	94.6 (86.2-98.0)	95.2 (87.6-98.2)	95.6 (88.7-98.4)
Other^c^									
Yes	1.1 (0.2-6.1)	0.4 (0.0-2.6)	0.6 (0.1-4.2)	0.0	0.0	1.3 (0.2-6.9)	1.4 (0.3-7.6)	1.3 (0.2-6.8)	1.2 (0.2-6.2)
No	98.9 (93.9-99.8)	99.6 (97.4-100.0)	99.4 (95.8-99.9)	100.0	100.0	98.7 (93.1-99.8)	98.6 (92.4-99.7)	98.7 (93.2-99.8)	98.8 (93.8-99.8)

APP, advanced practice provider; CI, confidence interval; RN, nurse.

a Housing instability, food insecurity, transportation difficulties, trouble paying for utilities, intimate partner violence, other exposure to violence, substance use, or mental health conditions.

b Unweighted frequencies are shown.

c “Other” included documentation with paper, a screening tool, and a social worker database.

**Table 3:** Association of emergency department characteristics with the presence of adverse social drivers of health documentation policies.

ED characteristics	OR (95% CI)^a^
Academic	0.62 (0.21-1.83)
Urban	0.60 (0.22-1.67)
Total ED visits	
<10,000	Reference
10,000-19,999	1.16 (0.33-4.04)
20,000-39,999	1.81 (0.52-6.34)
≥40,000	3.08 (0.90-10.58)
Social work services are available in the ED 24 h/d, 7 d/wk	2.86 (1.09-7.52)

ED, emergency department; OR, odds ratio.

a From survey-weighted logistic regression model.

### 3.1 Limitations

Limitations include a lack of specification regarding how aSDOH were documented within the EHR and, among EDs screening for multiple risk factors, which documentation methods were applied to specific needs. Given our response rate was 83%, we also acknowledge the possibility of response bias, although we attempted to mitigate this by performing stratified random sampling.

## 4 Discussion

In this national study of US EDs, over 90% of EDs that had aSDOH screening policies also had documentation policies. These policies typically assigned the responsibility of documenting aSDOH to clinical providers and expected EHR entry. Documentation requirements were highest for regulatory-required screenings (eg, violence, substance use, and mental health). The presence of 24/7 social work services was associated with greater odds of having an aSDOH documentation policy.

There is an ongoing debate about who should be responsible for screening and documenting aSDOH in EDs. Although ED leaders have emphasized the need for dedicated staff, particularly social workers,^6^ our findings show that this responsibility typically falls on clinical providers. This is problematic, as documenting aSDOH may not be the best use of providers’ time given their primary clinical duties and could contribute to burnout.^7^ We found that hospitals with 24/7 social work coverage were more likely to have at least 1 aSDOH documentation policy, suggesting either that social workers handle positive screens or that better-resourced hospitals emphasize documentation more.^8^ However, even in hospitals with social workers, high patient volumes—especially in facilities serving vulnerable populations—may make it impossible for social workers to evaluate every patient with aSDOH. Emerging ambient scribe technology could help by reducing the documentation burden, allowing clinicians and staff to focus more on interventions.

Among EDs requiring documentation of aSDOH, 100% required EHR documentation. This is inconsistent with studies showing underdocumentation.^2^^,^^3^ The data may be entered in a separate field (eg, nursing flowsheet) or simply not documented, despite requirements, indicating a need for standardized approaches.

Our findings demonstrate that while documentation of positive aSDOH screens is common practice in US EDs and universally occurs within the EHR, this responsibility predominantly falls to clinical providers rather than dedicated staff. Although this widespread documentation practice is encouraging, the lack of standardization in how this information is captured and utilized represents a critical gap that must be addressed to effectively translate aSDOH data into meaningful interventions and support population health initiatives. Future research should examine specific documentation workflows, evaluate the quality and accessibility of captured aSDOH data, and identify strategies to optimize both the documentation process and the subsequent use of this information to improve emergency care delivery for socially vulnerable patients.

## Author Contributions

MSK, CAC and MM conceptualized the study. KMB performed data collection. JAE analyzed the data, with REC performing a critical review and evaluation of the results. MFM primarily authored the paper, with all authors conducting final reviews and edits.

## Funding and Support

This study was supported by a 10.13039/100007619Society for Academic Emergency Medicine Foundation Research Large Project Grant. The funder had no role in the design and conduct of the study; collection, management, analysis, and interpretation of the data; preparation, review, or approval of the manuscript; and decision to submit the manuscript for publication.

## Conflict of Interest

Dr. Margaret Samuels-Kalow reports financial support was provided by Society for Academic Emergency Medicine. If there are other authors, they declare that they have no known competing financial interests or personal relationships that could have appeared to influence the work reported in this paper.
